# Mitral Regurgitation and Body Mass Index Increase the Predictability of Perioperative Bleeding in Anticoagulated Patients With Nonvalvular Atrial Fibrillation

**DOI:** 10.3389/fcvm.2022.846590

**Published:** 2022-03-28

**Authors:** Hao Huang, Chi Cai, Wei Hua, Nixiao Zhang, Hongxia Niu, Xuhua Chen, Jing Wang, Yuhe Jia, Jianmin Chu, Min Tang, Shu Zhang

**Affiliations:** ^1^State Key Laboratory of Cardiovascular Disease, Department of Cardiology, Fuwai Hospital, National Center for Cardiovascular Diseases, Chinese Academy of Medical Sciences and Peking Union Medical College, Beijing, China; ^2^Department of Cardiology, Cardiovascular Center, Beijing Friendship Hospital, Capital Medical University, Beijing, China

**Keywords:** atrial fibrillation, mitral regurgitation, body mass index, bleeding risk assessment, ABC bleeding score, HAS-BLED score

## Abstract

**Background:**

Catheter ablation (CA) effectively restores sinus rhythm in atrial fibrillation (AF) but causes a short-term fluctuation in the coagulation state. Potential risk factors and better management during this perioperative period remain understudied.

**Methods:**

We consecutively included 940 patients with nonvalvular AF who received CA at Fuwai Hospital, Beijing, China. Patients were divided into two groups according to their bleeding status during 3 months' anticoagulation. Any adverse events related to bleeding in the 3 months were evaluated. The HAS-BLED score and ABC-bleeding score, as well as other potential factors, were explored to predict bleeding risk.

**Results:**

In this observational study, 8.0% and 0.9% of the whole population suffered from bleeding and thromboembolic events, respectively. After adjusting for known factors related to bleeding, mitral regurgitation (MR, p for trend <0.001) and body mass index (BMI, odds ratio (OR) = 0.920, 95% CI 0.852–0.993, *p* = 0.033) were the most significant ones. C-indexes of the HAS-BLED score and ABC-bleeding score for bleeding were 0.558 (0.492–0.624) and 0.585 (0.515–0.655), respectively. The incorporation of MR and BMI significantly improved the predictive value based on HAS-BLED score (C-index = 0.650, 95% CI 0.585–0.715, *p* = 0.004) and ABC-bleeding score (C-index = 0.671, 95% CI 0.611–0.731, *p* < 0.001). The relative risk of mild-moderate MR was 4.500 (95% CI 1.625–12.460) in patients with AF having HAS-BLED = 1 and 4.654 (95% CI 1.496–14.475) in HAS-BLED ≥ 2, while it was not observed in patients with HAS-BLED = 0 (*p* = 0.722).

**Conclusion:**

More severe MR and lower BMI are associated with a higher incidence of perioperative bleeding, which helps improve the predictability of increased individual bleeding risk of a patient with nonvalvular AF who has received CA therapy and oral anticoagulants.

## Introduction

Atrial fibrillation (AF) is the most common serious abnormal heart rhythm, affecting millions of people worldwide and leading to substantial morbidity and mortality (1). Catheter ablation (CA), as an effective treatment modality of AF and atrial fibrillation flutter, is now gaining increasing traction. However, the procedure can entail serious complications, including stroke, transient ischemic attack, myocardial, and any other ischemic events (2). As a result, prevention of stroke and systemic thromboembolism remains the cornerstone for the perioperative population.

For over six decades, traditional anticoagulants, including low-molecular-weight heparin and vitamin K antagonists (VKAs), have been used to achieve therapeutic anticoagulation (3). In the past decade, given the profile of efficacy, safety, and convenience compared to VKAs (4), direct oral anticoagulants (DOACs) have gradually dominated clinical practice, as well as in patients with AF undergoing ablation (5). Nonetheless, decision-making for anticoagulation therapy depends on the assessment of both thromboembolic and bleeding risks. Current guidelines recommend that systemic anticoagulation with warfarin or a DOAC is continued for at least 2 months post ablation, and long-term continuation of systemic anticoagulation beyond 2 months is based on the stroke risk profile of the patient and not on the apparent success or failure of the ablation procedure (6). The specific duration of oral anticoagulants' (OACs) use and potential risk factors related to bleeding during this period remain inconclusive.

Several bleeding scoring systems, including HAS-BLED, HEMORR2HAGES, anemia (3 points), severe renal disease (3 points), age ≥ 75 years (2 points), prior bleeding (1 point), and hypertension (1 point) (ATRIA), and older [age ≥ 74 years], reduced hemoglobin/hematocrit/history of anemia, bleeding history, insufficient kidney function, and treatment with antiplatelet (ORBIT), designed for predicting long-term bleeding risk have been established and validated in patients with AF (7–10), among which HAS-BLED has the best evidence for predicting bleeding risk (moderate strength of evidence) confirmed by systematic reviews and meta-analyses (11–13). The biomarker-based ABC-bleeding risk score reported outperformed clinical scores in some studies (14, 15), but its predictability is still in debate, especially in identifying patients at low risk of bleeding (12, 16), who exactly constitute a large proportion of candidates predisposing to CA in an early stage of AF. The *post-hoc* analyses of clinical trials and many observational studies include patients with anticoagulated AF with a higher thromboembolic risk, mostly with CHA2DS2-VASc score ≥ 2, so bleeding score evaluation has been mostly applied to this demographic. However, patients initially admitted for CA usually share a less severe thromboembolic tendency. In contrast, many patients with AF experience dynamic bleeding risk in this perioperative period, which indicates that the current dose and duration of OACs might cause bothering bleeding. The change in bleeding risk profile is a stronger predictor of major bleeding events, especially in the first 3 months (17). Better management to identify patients at risk of bleeding who should prudently use OACs or patients at risk of thromboembolic (TE) events who should continue OACs after CA still needs to be improved. Therefore, this study aimed to evaluate the most two acknowledged bleeding risk scores, HAS-BLED score and ABC-bleeding score, and explore other potential bleeding risk factors in this specific proportion of patients with AF who had undergone CA in a large-scale observational case-control study in the Asian population.

## Methods

### Study Population

**Figure 1:**
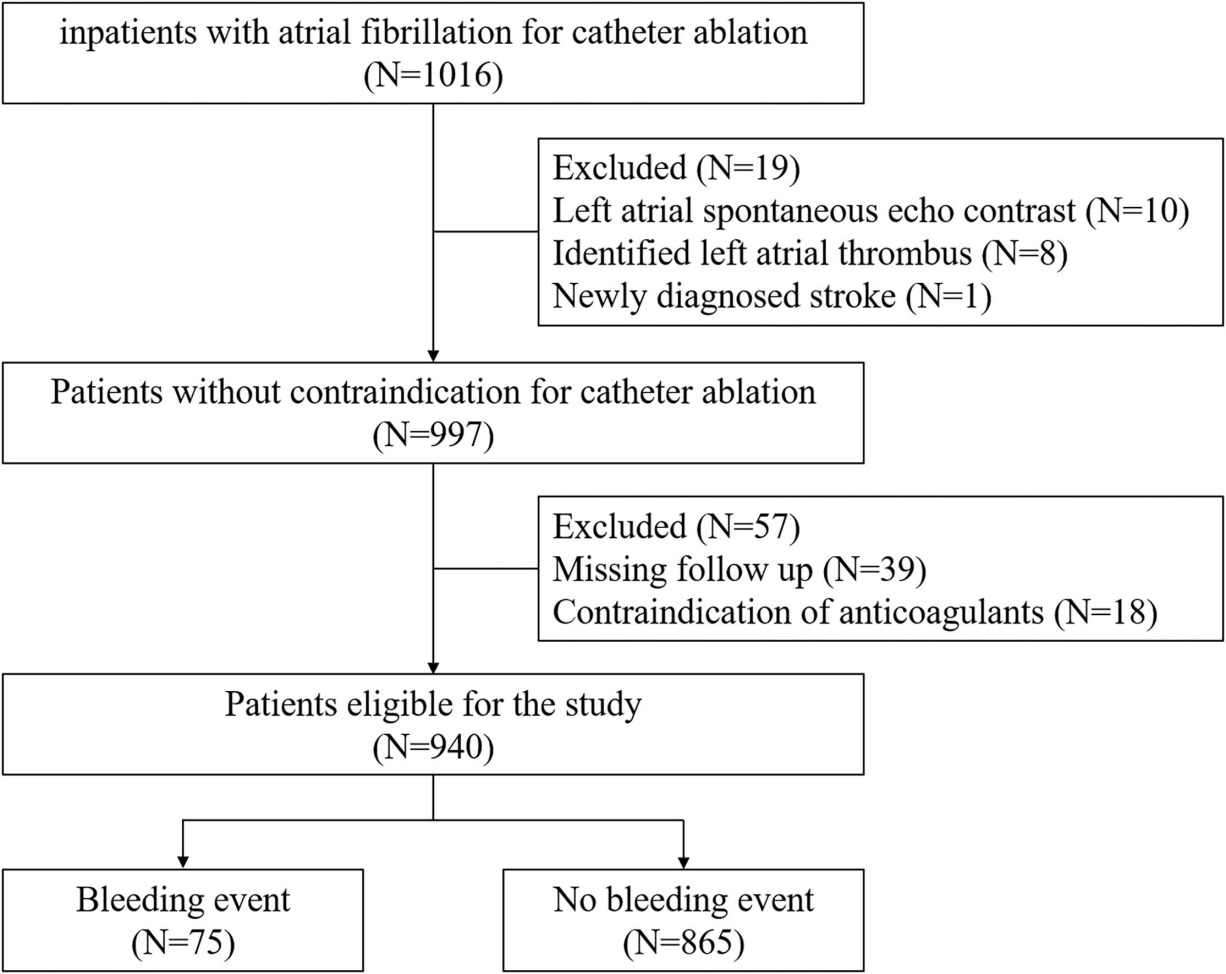
Patient flow diagram.

The patients' flow program is shown in Figure 1. In this single-center, retrospective, observational study, the inclusion criterion was age ≥ 18, documentation of AF on 12-lead electrocardiogram or Holter monitor. We enrolled 1,016 consecutive patients intended for CA at Fuwai Hospital between June 2017 and March 2019. Patients with signs of left atrial spontaneous echo contrast or identified thrombus or newly diagnosed stroke were excluded (*n* = 19). Those with contraindication for anticoagulant use were also excluded (*n* = 18). A total of 39 patients were lost to follow-up within 90 days after ablation. Finally, the remaining 940 participants were divided into two groups, that is, patients with (*n* = 75) or without bleeding events (*n* = 865). All procedures were conducted in accordance with the Declaration of Helsinki and its amendments. The study protocol was approved by the human ethics committee of Fuwai Hospital, and written informed consent was obtained from each patient or the patient's family.

### Periprocedural Anticoagulant Management

Patients were required to receive continuous oral anticoagulation for at least 3 weeks before ablation if AF had lasted for over 48 h or uncertain time, and was named delayed CA strategy. Early CA strategy of anticoagulation for 1–7 days was employed if immediate transesophageal echocardiography or contrast-enhanced CT verified the absence of an intracardiac thrombus. The choice of DOACs or warfarin was determined by both the attending physician and the patient. The DOACs included dabigatran and rivaroxaban. The warfarin dose was strictly adjusted to maintain a target international normalized ratio of 2.0–3.0 before and 3 months after CA. Anticoagulants were continued during the procedure. No heparin bridging was performed. On the procedure day, warfarin and dabigatran were administered in the morning and evening at routine doses, while rivaroxaban was administered only in the morning at routine doses. Most patients were recommended to take OACs, along with one type of antiarrhythmic drug and proton pump inhibitors that were continuously administered for at least 3 months unless severe adverse events happened. After the procedure, dabigatran was administered in the morning and evening at 110 mg, while rivaroxaban was administered only in the morning at 20 or 15 mg in a population with creatinine clearance (CrCl) between 30 and 49 ml/min.

### Ablation

All procedures were performed under conscious sedation. Vascular access was obtained *via* the right femoral and right subclavian veins. On completion of the transseptal puncture under fluoroscopic guidance, patients received intravenous heparin to maintain an activated clotting time of >300 s. The 3D endocardial surface of the LA and pulmonary veins was constructed under the guidance of the CARTO 3 system. Ablation techniques varied according to the operator's discretion, anatomical features, type of AF, and history of previous ablations. Techniques included ipsilateral pulmonary vein isolation (PVI) with a wide area of circumferential ablation, focal activity ablations, superior vena cava isolation, and atrial substrate modification by applying ablation at complex fractionated atrial electrograms, and/or additional LA linear ablation. During PVI, a circumferential mapping catheter (Lasso, Biosense Webster, CA, USA) was placed inside the ipsilateral PV. The endpoint of PVI was defined as the absence of any PV spike potential recorded on the Lasso catheter.

### Data Collection

All medical records included complete records of diagnoses, prescriptions, and results of laboratory examinations of inpatients. Therefore, the dataset was populated using medical records and/or medical information systems. The following laboratory data were included for analysis: CrCl, estimated glomerular filtration rate (eGFR) by the Chronic Kidney Disease Epidemiology Collaboration (CKD-EPI) equation, aspartate aminotransferase (AST), platelet count, hemoglobin, international normalized ratio (INR), N-terminal B-type natriuretic peptide (NT-proBNP), high-sensitive troponin I (hs-TnI), creatine, blood urea nitrogen (BUN), uric acid, high-sensitive C-reactive protein (hsCRP), and HbA1c. Samples of laboratory analysis for measurement were obtained before CA. Stroke risk was calculated using the CHA2DS2-VASc score [congestive heart failure, hypertension, age ≥ 75 years, diabetes mellitus, stroke or transient ischemic attack, vascular disease, age 65–74 years and sex category (female)] (18). Bleeding risk was calculated using the HAS-BLED score [Hypertension (uncontrolled systolic blood pressure > 160 mmHg), abnormal renal and/or liver function, stroke history, bleeding history or predisposition (anemia), labile INR (only applies to a VKA user; not applicable for a non-VKA user), elderly (age>65 years), and concomitant drugs (antiplatelet or nonsteroidal anti-inflammatory drugs) and/or alcohol excess] (8). Due to the difference in study design, outcome type, and follow-up time, the ABC-bleeding score was not calculated with the original coefficients, hence we evaluated the robustness of the original five factors, including age, bleeding history, hsTnI, hemoglobin, and CKD-EPI in this new cohort (14).

### Transthoracic Echocardiography

Each subject underwent transthoracic M-mode, 2D, and Doppler echocardiography using commercially available echocardiographic units (Vivid 7, GE Healthcare, Milwaukee, USA, or Philips SONOS 7500, Best, the Netherlands). Preoperative transthoracic echocardiography was obtained while the patients were at rest within 1 week before CA using a Philips IE33 ultrasound device (Philips Medical Systems, Andover, MA, USA). Two-dimensional echocardiographic measurements and Doppler evaluation were performed according to the American Society of Echocardiography guidelines (19). Five cardiac cycles were collected for analysis in patients with AF. The severity of mitral regurgitation (MR) was defined using a multiparametric approach, including an assessment of the color Doppler-derived jet area, the effective regurgitant orifice area using the proximal isovelocity surface area method, the MR volume and fraction using the Doppler-derived volumetric method, and the pulmonary vein flow velocity pattern (20). The tricuspid regurgitation (TR) was also defined using a multiparametric approach, including an assessment of the color Doppler-derived jet area, the continuous wave Doppler-derived jet density and contour, and the hepatic vein flow velocity pattern (20). For the statistical analysis in this study, the grading of MR or TR was graded as none, trace, mild, and moderate. None of the patients had severe MR or TR. Given the limited number of patients with moderate MR (5/940) and TR (7/940), mild/moderate MR or TR were classified as one grade.

### Outcomes

Every patient was followed up by the electronic medical record system or at least one telephone inquiry in 3 months after CA. Periprocedural bleeding events were defined as bleeding complications within 90 days after CA, which was further differentiated into procedure-related (bleeding around puncture point and cardiac tamponade) and unrelated events. Multiple bleeding happened in one person involving puncture site bleeding or cardiac tamponade and other bleedings would be accounted for once in total bleeding analysis, while twice in both procedure-related or unrelated bleeding analysis. Major bleeding events were defined as a reduction in hemoglobin by ≥2 g/dL, transfusion ≥2 units of blood, or symptomatic bleeding in a critical area or organ, such as cerebral or intraspinal hemorrhages, following the International Society on Thrombosis and Hemostasis definition (21). Minor bleeding was defined as overt bleeding not meeting the criteria for MB but requiring medical intervention, unscheduled contact (visit or telephone) with a physician, temporary interruption, or delayed dosing of the use of an anticoagulant, pain, or impairment of daily activities.

### Statistical Analysis

Categorical variables were expressed using percentages and continuous parameters using mean ± standard deviation or median (interquartile range). Kolmogorov–Smirnov test was used for normality test. Comparisons among the two groups were conducted using the one-way ANOVA or Mann-Whitney *U*-test for continuous variables and χ^2^ test or Fisher's exact test for dichotomous variables. Univariate logistic regression analysis was performed to identify the association between potential variables and perioperative bleeding. Variables with a *p* < 0.1 in univariate analysis were entered into the multivariate analysis. Candidate variables were selected using backward selection (entry criterion *p* = 0.05, removal *p* = 0.1) or Akaike information criterion (AIC) rule. HAS-BLED categories were defined as low/moderate risk (0–1 points) and high risk (≥2). ABC-bleeding risk categories were defined as low/moderate risk (0–7/7–10% predicted 3-month risk of bleeding) and high risk (>10% predicted risk of 3-month bleeding). CHA2DS2-VASc risk score categories were defined as low risk (male = 0, female = 1), moderate risk (male = 1, female = 2), and high risk (male ≥ 1, female ≥ 2) according to the recommendation of anticoagulant use in the 2020 ESC guideline (6). Calibration was assessed by comparing observed 3-month event rates with predictions from the adjusted models. Discrimination of different scores combined with additional variables was assessed by Harrell's C-index, integrated discriminatory improvement (IDI), and net reclassification improvement (NRI), and also in different subgroups: DOAC, CHA2DS2-VASc score low/moderate risk or moderate/high risk. Clinical usefulness and net benefit were estimated with decision curve analysis. All tests were two-sided, and *p* < 0.05 was considered statistically significant. All statistical analyses were performed with SPSS version 26.0 (Windows, SPSS, Inc., Chicago, IL, USA) and R version 4.0.3 (R Foundation for Statistical Computing, Vienna, Austria, 2008).

## Results

### Demographic and Clinical Characteristics Regarding Bleeding

Baseline characteristics of possible factors regarding bleeding in patients with AF during the perioperative period are summarized in Table 1. Total bleeding events represent 8.0% (75/940) of the whole population. Two major bleeding events occurred both as pericardial tamponade while most bleeding events were regarded as minor bleeding events. Totally, 7 patients suffered from multisite bleeding (Supplementary Table 1). There were no significant differences between the two groups regarding gender, medical history, perioperative history, and most known bleeding factors. However, patients with bleeding events were elderly (>75 years, 8.0 vs. 2.5%, *p* = 0.012) and underweight (25.09 ± 3.22 vs. 26.03 ± 3.30, *p* = 0.021). Among previously validated bleeding factors, kidney dysfunction defined as creatine clearance less than 50 ml/min was more prevalent (8.0 vs. 3.2%, *p* = 0.041) in the bleeding group. It is worth noting that the bleeding group was predisposed to more severe valvular regurgitation. The odds ratio (OR) of mild to moderate MR and trace MR was 3.556 (95% CI 1.882–6.721, *p* < 0.001) and 2.062 (95% CI 1.095–3.884, *p* = 0.025), respectively (*p* for trend <0.001). TR showed a similar trend, though the risk of trace regurgitation was not statistically significant (*p* = 0.063).

**Table 1:** Baseline characteristics of patients with AF regarding bleeding.

	**Total**	**Bleeding events**	**No bleeding events**	**Odds ratio**	***p*-Value**
	**(*n* = 940)**	**(*n* = 75)**	**(*n* = 865)**	**(95% CI)**	
Age (years)	58.02 ± 9.82	60.03 ± 10.46	57.85 ± 9.76	1.024 (0.998–1.050)	0.066
>65	219 (23.3)	24 (32.0)	195 (22.5)	1.617 (0.970–2.694)	0.065
≥75	28 (3.0)	6 (8.0)	22 (2.5)	3.332 (1.308–8.491)	0.012
Male sex	632 (67.2)	46 (61.3)	586 (67.7)	0.755 (0.464–1.228)	0.258
BMI (kg/m^2^)	25.96 ± 3.30	25.09 ± 3.22	26.03 ± 3.30	0.915 (0.848–0.987)	0.021
Duration since first diagnosed (years)	2.00 (0.50, 5.00)	1.75 (0.42, 5.00)	2.00 (0.50, 5.00)	0.989 (0.939–1.041)	0.668
Medical history					
Paroxysmal AF	549 (58.4)	48 (64.0)	501 (57.9)	1.292 (0.791–2.109)	0.306
Hypertension	516 (54.9)	46 (61.3)	470 (54.3)	1.333 (0.822–2.162)	0.244
Diabetes mellitus	184 (19.6)	13 (17.3)	171 (19.8)	0.851 (0.457–1.583)	0.610
Coronary artery disease	153 (16.3)	16 (21.3)	137 (15.8)	1.441 (0.805–2.578)	0.218
LV dysfunction	18 (1.9)	1 (1.3)	17 (2.0)	0.674 (0.088–5.136)	0.703
Stroke/TIA	121 (12.9)	10 (13.3)	111 (12.8)	1.045 (0.522–2.094)	0.901
Myocardial infarction	29 (3.1)	3 (4.0)	26 (3.0)	1.345 (0.397–4.550)	0.634
CHA2DS2-VAaSc score	1 (1,3)	2 (1,3)	1 (1,3)	1.110 (0.956–1.290)	0.172
Perioperative history					
Intraoperative heparin (IU)	9,000 (7,000, 10,000)	8,500 (7,000, 10,000)	9,000 (7,000, 10,000)	0.981 (0.904–1.063)	0.636
Preoperative anticoagulation History	469 (49.9)	37 (49.3)	432 (49.9)	0.976 (0.609–1.564)	0.919
Preoperative anticoagulation time (month)	0 (0, 1)	0 (0, 1)	0 (0, 1)	1.009 (0.994–1.025)	0.251
Oral anticoagulant					
Warfarin	135 (14.4)	9 (12.0)	126 (14.6)	Reference	Reference
DOACs	805 (85.6)	66 (88.0)	739 (85.4)	1.250 (0.608–2.573)	0.544
Bleeding risk factors					
Antiplatelet drugs	11 (1.2)	0 (0.0)	11 (1.3)	0.000 (0.000–∞)	0.999
Previous bleeding	10 (1.1)	1 (1.3)	9 (1.0)	1.285 (0.161–10.284)	0.813
SBP at entry	127.6 ± 15.9	129.0 ± 14.8	127.5 ± 16.0	1.062 (0.919–1.228)	0.413
CrCl <50 mL/min	34 (3.6)	6 (8.0)	28 (3.2)	2.599 (1.041–6.492)	0.041
eGFR <60 mL/min/1.73 m^2^ (CKD-EPI)	87 (9.3)	11 (14.7)	76 (8.8)	1.784 (0.902–3.528)	0.096
Smoking	335 (35.6)	28 (37.3)	307 (35.5)	1.083 (0.665–1.764)	0.749
Alcohol use	268 (28.5)	26 (34.7)	242 (28.0)	1.366 (0.830–2.248)	0.220
Anemia	23 (2.4)	2 (2.7)	21 (2.4)	1.101 (0.253–4.789)	0.898
INR at entry	1.02 (0.96,1.16)	1.03 (0.97, 1.17)	1.02 (0.96, 1.16)	0.937 (0.542–1.622)	0.817
MR					
None	744 (79.1)	46 (61.3)	698 (80.7)	Reference	Reference
Trace	117 (12.4)	14 (18.7)	103 (11.9)	2.062 (1.095–3.884)	0.025
Mild–moderate	79 (8.4)	15 (20.0)	64 (7.4)	3.556 (1.882–6.721)	<0.001
p for trend					<0.001
TR					
None	737 (78.4)	49 (65.3)	688 (79.5)	Reference	Reference
Trace	133 (14.1)	15 (20.0)	118 (13.6)	1.785 (0.969, 3.286)	0.063
Mild-moderate	70 (7.4)	11 (14.7)	59 (6.8)	2.618 (1.292, 5.303)	0.008
*p* for trend					0.011
LAD (mm)	39.9 ± 5.4	40.4 ± 4.9	39.8 ± 5.4	1.022 (0.979–1.067)	0.326
LVEDD (mm)	48.0 ± 4.7	47.5 ± 4.7	48.0 ± 4.7	0.973 (0.924–1.025)	0.308
Other biomarkers					
NT-proBNP (pg/mL)	178.7 (66.3, 543.6)	217.7 (76.7, 593.4)	176.6 (64.5, 533.9)	1.000 (1.000–1.001)	0.072
hs–TnI (ng/L)	2.0 (0.0, 4.0)	3.0 (0.0,6.0)	2.0 (0.0,4.0)	1.001 (1.000–1.003)	0.072
Platelet (10^9^/L)	223 (191, 262)	221 (181, 246)	224 (192, 262)	0.997 (0.993–1.002)	0.216
Hemoglobin (g/L)	155 (144, 165)	151 (141, 163)	155 (144, 166)	0.988 (0.974–1.003)	0.110
AST (U/L)	21 (18, 25)	21 (17, 23)	21 (18, 25)	0.969 (0.932, 1.008)	0.114
Creatine (μmol/L)	81.34 (72.56, 91.77)	78.49 (72.02, 90.12)	81.89 (72.56, 91.79)	0.998 (0.983–1.015)	0.850
BUN (mmol/L)	5.50 (4.70, 6.50)	5.40 (4.50, 6.50)	5.50 (4.70, 6.55)	1.011 (0.864–1.182)	0.892
Uric acid (μmol/L)	346.3 (291.8, 407.9)	348.9 (278.9, 407.4)	345.9 (291.8, 408.0)	1.000 (0.997–1.003)	0.975
hsCRP (mg/L)	1.19 (0.58, 2.23)	1.58 (0.77, 2.36)	1.17 (0.57, 2.19)	1.015 (0.919–1.121)	0.772
HbA1c (%)	5.85 (5.50, 6.30)	5.90 (5.60, 6.40)	5.80 (5.50, 6.30)	1.113 (0.871–1.423)	0.392

*Data are given as median (25th percentile, 75 percentile) or n (%). AST, aspartate aminotransferase; BMI, body mass index; BUN, blood urea nitrogen; CrCl, creatinine clearance; DOAC, direct oral anticoagulants; eGFR, estimated glomerular filtration rate; hs-CRP, high-sensitive C-reactive protein; hs-TnI, high-sensitive troponin I; INR, international normalized ratio, LAD, left atrial diameter; LVEDD, left ventricular end-diastolic diameter; MR, mitral regurgitation; NSAIDs, nonsteroidal anti-inflammatory drugs; NT-proBNP, N-terminal B-type natriuretic peptide; SBP, systolic blood pressure; TIA, transient ischemic attacks; TR, tricuspid regurgitation*.

### Development and Validation of the Model for Predicting Perioperative Bleeding Events

Given the inclusion rule mentioned above, age, body mass index (BMI), MR, TR, creatine clearance, NT-proBNP, and hs-TnI were entered into multivariate analysis (Table 2). After backward selection of these variables or based on the minimum AIC value, MR and BMI were the most two important predictors. Compared with patients without MR, those with trace and mild/moderate MR are 2.067 (95% CI 1.095–3.902, *p* = 0.025) and 3.415 (95% CI 1.802–6.474, *p* < 0.001) times more likely to suffer from bleeding (p for trend <0.01). BMI was negatively associated with bleeding. For each unit increase, the risk of bleeding decreased to 0.920 (95% CI 0.852–0.993, *p* = 0.033). In order to testify the robustness of this finding, Supplementary Table 2 shows the variables correlated to procedure-related or procedure-unrelated bleeding. It was indicated that MR maintained a significant association with both outcomes, especially for mild-moderate MR. Higher BMI was associated with a lower rate of procedure-unrelated bleeding, while no effect was seen on procedure-related bleeding. Therefore, MR and BMI were selected to be incorporated into traditional bleeding risk scores.

**Table 2:** Univariate and multivariate logistic regression model of possible indicators for periprocedural bleeding events.

**Characteristics**	**Unadjusted OR (95% CI), *p*-value**	**Adjusted OR (95% CI)**, ***p*****-value**	
		**Model 1**	**Model 2**
Age	1.024 (0.998–1.050), 0.066	1.018 (0.984–1.052), 0.302	
BMI	0.915 (0.848–0.987), 0.021	0.925 (0.844–1.013), 0.093	0.920 (0.852–0.993), 0.033
MR			
None	Reference	Reference	Reference
Trace	2.062 (1.095–3.884), 0.025	1.713 (0.810–3.622), 0.159	2.067 (1.095–3.902), 0.025
Mild-moderate	3.556 (1.882–6.721), <0.001	3.251 (1.355–7.800), 0.008	3.415 (1.802–6.474), <0.001
*p* for trend	<0.001	0.025	0.001
TR			
None	Reference	Reference	
Trace	1.785 (0.969, 3.286), 0.063	1.189 (0.580–2.436), 0.636	
Mild-moderate	2.618 (1.292, 5.303), 0,008	0.918 (0.341–2.472), 0.951	
*p* for trend	0.011	0.847	
CrCl (mL/min)	0.990 (0.980–0.999), 0.034	1.001 (1.000–1.001), 0.931	
NT-proBNP (pg/mL)	1.000 (1.000–1.001), 0.072	1.000 (1.000–1.001), 0.196	
hs-TnI (ng/L)	1.001 (1.000–1.003), 0.072	1.001 (1.000–1.003), 0.123	

*Abbreviations as mentioned in Table 1*.

The distribution of bleeding events according to bleeding risk score categories is shown in Table 3. Of the study cohort, 152 (16.2%) were categorized as “high risk” using the HAS-BLED score compared to 142 (15.1%) with the ABC-bleeding score. The categorized HAS-BLED score (*p* = 0.046) and ABC score (*p* = 0.021) were both able to differentiate bleeding events. As shown in Table 4, these two risk scores showed C-index values of 0.58 (95% CI 0.49–0.63) and 0.58 (95% CI 0.51–0.65), respectively. No difference was found between them (*p* = 0.399). Adding MR status into the ABC score achieved a higher C-index (0.652 vs. 0.585, *p* = 0.031), while the effect was not significant when adding into the HAS-BLED score (0.61 vs. 0.56, *p* = 0.065). NRI analyses showed a significant positive reclassification (38.72 %, *p* < 0.001; 39.41%, *p* < 0.001) and also a significant positive IDI (2.1%, *p* < 0.001; 1.73%, *p* = 0.003) when adding MR into the HAS-BLED score or the ABC-bleeding score compared to the two original scores alone. Combining both MR and BMI (fully adjusted), on the other hand, improved the performance of both risk bleeding scores based on the C-index (0.65 vs. 0.58, *p* = 0.005; 0.67 vs. 0.58, *p* = 0.013), NRI (38.89%, *p* = 0.001; 46.09%, *p* < 0.001), and IRI (2.43%, *p* < 0.001; 2.01%. *p* = 0.001). The internal 1,000 bootstrap samples validation indicated the corrected C-index values of 0.634 and 0.629 in both additional scores. BMI alone did not improve the predictability of ABC score, while it slightly improved HAS-BLED score only based on IDI (0.59%, *p* = 0.026). A sensitivity analysis for procedure-unrelated bleeding showed similar results (Supplementary Table 3).

**Table 3:** Distribution of bleeding events according to HAS-BLED, ABC and CHA2DS2-VASc score categories.

	**Risk category**	**No bleeding events**	**Bleeding events**	***p*-Value**
HAS-BLED score	0 (*n* = 442, 47.0%)	412 (47.6)	30 (40.0)	0.046
	1 (*n* = 346, 36.8%)	320 (37.0)	26 (27.6)	
	≥2 (*n* = 152, 16.2%)	133 (15.4)	19 (25.3)	
ABC score	Low risk (*n* = 344, 36.6%)	323 (37.3)	21 (28.0)	0.021
	Moderate risk (*n* = 454, 48.3%)	418 (48.3)	36 (48.0)	
	High risk (*n* = 142, 15.1%)	124 (14.3)	18 (24.0)	
CHA2DS2-VASc score	Low risk (*n* = 258, 27.4%)	240 (27.7)	18 (24.0)	0.157
	Moderate risk (*n* = 315, 33.5%)	295 (34.1)	20 (26.7)	
	High risk (*n* = 367, 39.0%)	330 (38.2)	37 (49.3)	

**Table 4:** C-indices, ROC curves comparison, IDI, and NRI of the HAS-BLED score and ABC-bleeding score with or without mitral regurgitation or BMI to predict perioperative bleeding in the full cohort.

**Full cohort**	**C-index (95% CI)**	***p*-Value**	**NRI (%, 95% CI)**	***p*-Value**	**IDI (%, 95% CI)**	***p*-Value**
HAS-BLED	0.58 (0.49-0.63)		Ref.		Ref.	
HAS-BLED + MR^†^	0.61 (0.54–0.68)	0.066	38.72 (16.06–61.38)	<0.001	2.1 (0.86–3.34)	<0.001
HAS-BLED + BMI^†^	0.59 (0.52–0.66)	0.319	23.23 (−0.03–46.49)	0.050	0.59 (0.08–1.09)	0.026
HAS-BLED + MR + BMI^†^	0.65 (0.58–0.71)	0.005	38.89 (15.49–62.29)	0.001	2.43 (1.13–3.73)	<0.001
						
ABC^†^	0.58 (0.51–0.65)	0.399	6.2 (−17.38–29.77)	0.606	1.07 (−0.08–2.96)	0.264
ABC + MR^※^	0.65 (0.59–0.72)	0.031	39.41 (16.76–62.07)	<0.001	1.73 (0.59–2.88)	0.003
ABC + BMI^※^	0.61 (0.54–0.68)	0.339	21.03 (−2.33–44.38)	0.078	0.41 (−0.03–0.85)	0.065
ABC + MR + BMI^※^	0.67 (0.61–0.73)	0.013	46.09 (22.69–69.49)	<0.001	2.01 (0.77–3.24)	0.001

*Data are C-indices, integrated discriminatory improvement (IDI), and net reclassification improvement (NRI) for each score and p-value for their comparison with the HAS-BLED score and ABC-bleeding score alone*.

*^†^p for comparing with HAS-BLED score*.

*^※^p for comparing with ABC score*.

**Figure 2:**
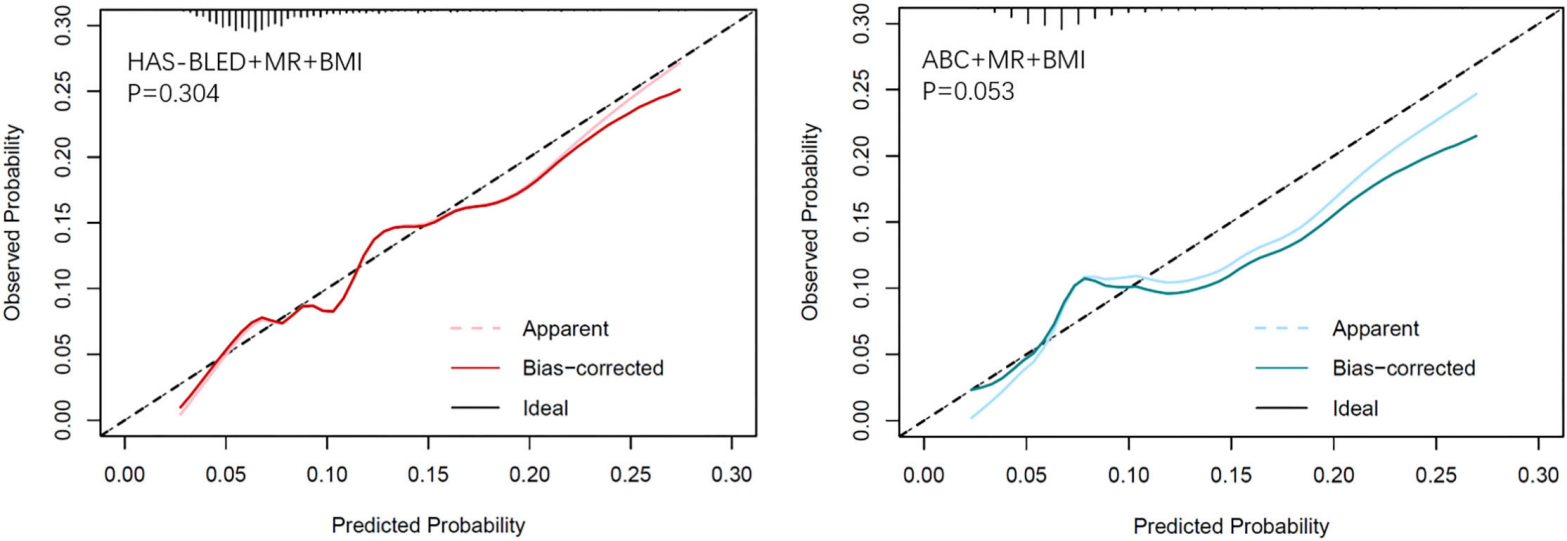
Calibration plots of observed vs. predicted event rate for the MR and BMI combined with HAS-BLED (left) and ABC-bleeding (right) risk models.

**Figure 3:**
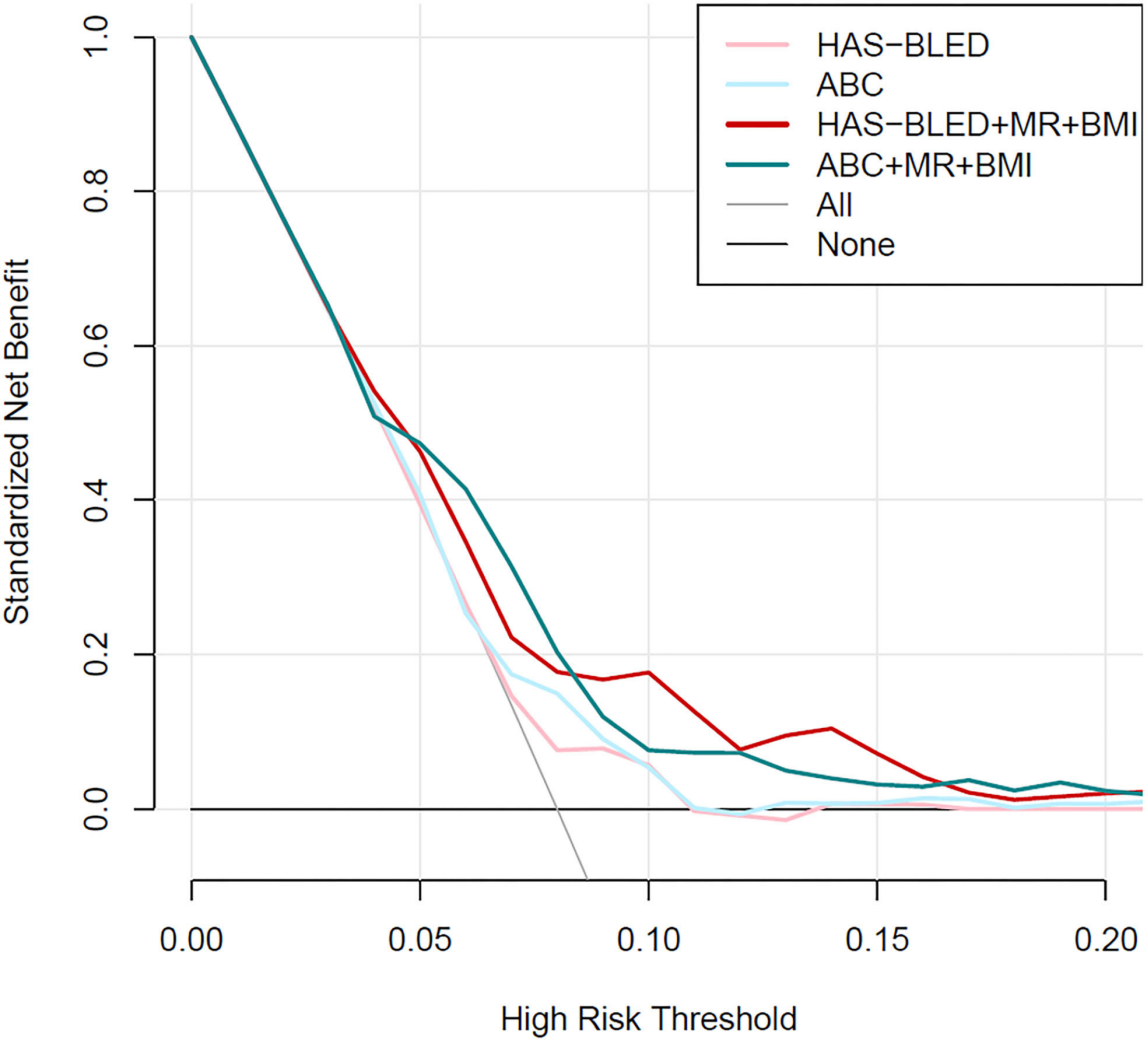
Decision curve analysis for the HAS-BLED and ABC-bleeding risk prediction models combined with mitral regurgitation and body mass index or not.

Calibration plots (Figure 2) indicated that both additional bleeding scores could well fit bleeding risks, although the ABC score combined with MR and BMI tended to underestimate patients with a high bleeding risk (>10%). Decision curve analysis was used to facilitate the comparison between different prediction models. As shown in Figure 3, the decision curve analysis graphically shows the clinical usefulness of each model based on a continuum of potential thresholds for bleeding risk (x-axis) and the standardized net benefit of using the model to risk-stratify patients (y-axis) relative to assuming that no patient will have bleeding events. In this analysis, the risk score combined with MR and BMI provided a larger net benefit across the range of bleeding risk compared with HAS-BLED or ABC score alone. For example, at a threshold of 8% perioperative bleeding risk, the HAS-BLED score combined with MR and BMI will identify 10.2% additional bleedings compared with using the HAS-BLED score alone, without increasing the number of false positives (Supplementary Table 4).

**Figure 4:**
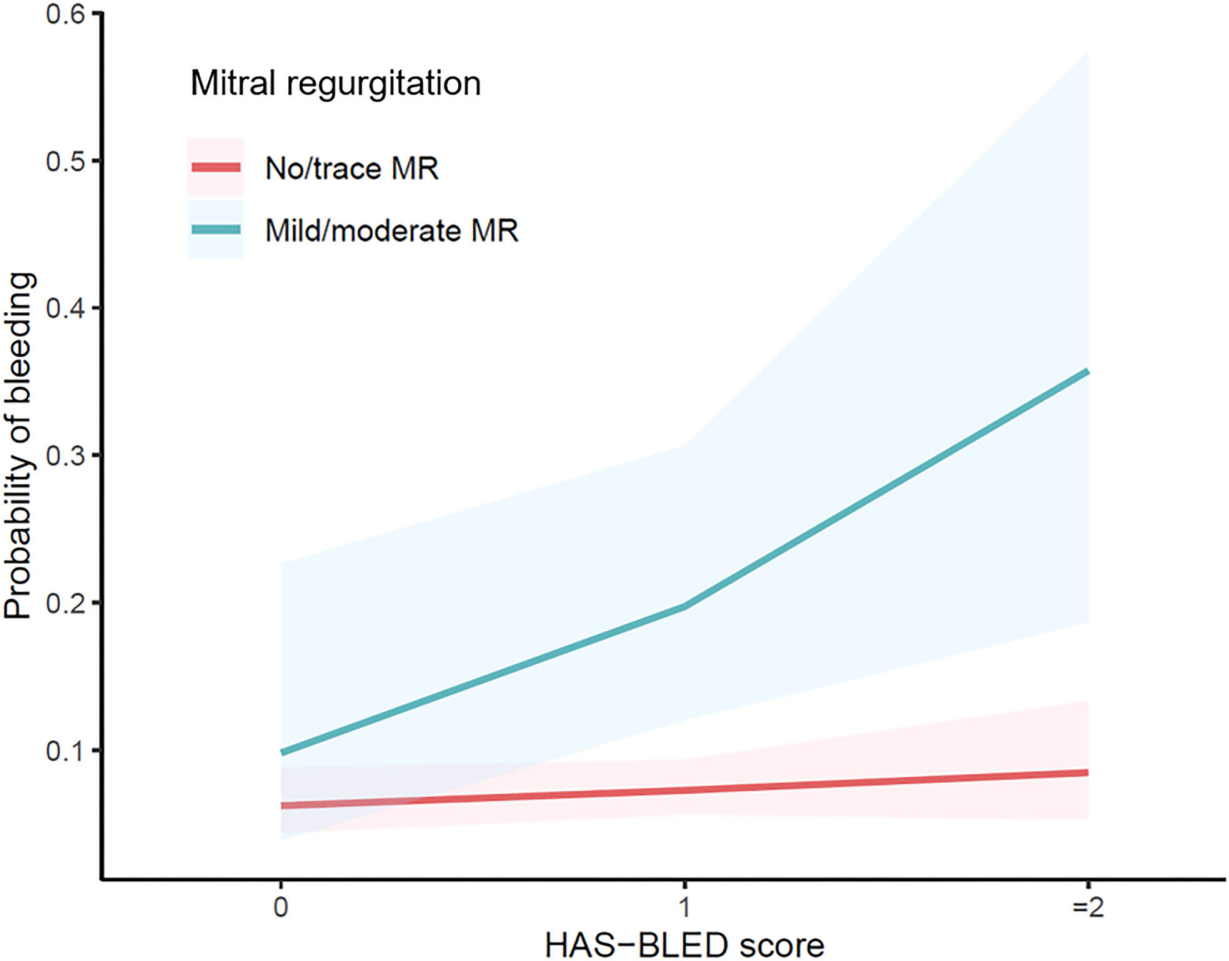
Odds ratio of different risk categories based on the HAS-BLED score with different mitral regurgitation status.

To further investigate the possible confounders of difference in bleeding risk of MR, variables with *p* < 0.1 in univariate analysis, along with postoperative OAC choice and echocardiographic markers, were compared between no/trace MR and mild/moderate MR. Patients in the latter group were older, had larger left atria and more severe TR, higher levels of NT-proBNP and hs-TnI, and lower levels of creatine clearance. However, no interaction was detected between these variables and MR (Supplementary Table 5). When dividing the full cohort into different thromboembolic and bleeding risk categories based on the HAS-BLED score and CHA2DS2-VASc score, the effect of MR seemed changed. The HAS-BLED score outperformed in patients with mild-moderate MR rather than none/trace MR (*p* < 0.001, Figure 4). Accordingly, MR remained a risk factor in patients with HAS-BLED ≥ 1 (OR = 4.500 in HAS-BLED = 1; *p* = 0.008 and OR = 4.654 in HAS-BLED ≥ 2, *p* = 0.012, Table 5) but not in HAS-BLED = 0 (*p* = 0.722). The interaction effect was not detected between MR and ABC risk categories. Besides, the ORs of mild/moderate MR were 3.600 and 3.758 in patients with CHA2DS2-VASc score moderate and high-risk groups while not significant in the low-risk group (Table 5).

**Table 5:** Odds ratio of mitral regurgitation (no/trace MR vs. mild/moderate MR) by different subgroups based on the CHA2DS2-VASc score and HAS-BLED score.

**Bleeding events**	**No/trace MR**	**Mild/moderate MR**	**Odds ratio**	***p*-Value**
	**(*n* = 861)**	**(*n* = 79)**	**(95% CI)**	
All (*n* = 940)	60 (7.0)	15 (19.0)	3.129 (1.682–5.819)	<0.001
HAS-BLED				<0.001
0 (*n* = 442)	27 (6.6)	3 (8.6)	1.319 (0.379–4.588)	0.662
1 (n = 346)	20 (6.3)	6 (23.1)	4.500 (1.625–12.460)	0.008
≥2 (*n* = 152)	13 (9.7)	6 (33.3)	4.654 (1.496–14.475)	0.012
CHA2DS2-VASc score				<0.001
Low risk (*n* = 258)	17 (7.0)	1 (7.1)	1.027 (0.127–8.328)	1.000
Moderate risk (*n* = 315)	19 (5.3)	5 (16.7)	3.600 (1.208–10.728)	0.031
High risk (*n* = 367)	28 (8.4)	9 (25.7)	3.758 (1.605–8.803)	0.004

## Discussion

Using real-world data from routine clinical practice, our study validated the most commonly used two bleeding scores and explored potential clinical bleeding factors in patients with nonvalvular AF (NVAF) after CA. The HAS-BLED score and ABC-bleeding risk score showed similar but relatively low predictability in this demographic. We found that two factors, MR and BMI, were associated with perioperative bleeding events, which added further predictability based on the HAS-BLED and ABC scores.

Although there is some variability in the periprocedural OAC management in patients undergoing AF ablation, more recently operators have moved toward a strategy of performing the ablation under uninterrupted VKA or DOAC treatment (22). In a US cohort of patients with AF who underwent CA followed for a median of 1.2 years, approximately a quarter of thromboembolic events occurred within the first 3 months post ablation (23). In the immediate post-ablation period, consensus supports OACs for at least 2–3 months due to an increased thrombotic risk from post-ablation inflammation and delayed recovery of atrial function (24). Additionally, there was an eightfold risk of thromboembolism following premature discontinuation of OACs within the first 3 months of ablation compared to patients who continued OACs during the same time period (25). Therefore, our study focused on this period when all the patients are intensively prescribed OAC, thus guaranteeing a fully anticoagulated background. We noticed that nearly half of the patients did not receive OACs before the operation, given that 64.7% of men had a CHA2DS2-VASc score ≤ 1 and 53.2% of women ≤ 2 in this cohort planning to receive CA. Among these populations, the original HAS-BLED and ABC-bleeding scores underperformed, thus requiring more sensitive predictors explored in this study.

According to the American College of Cardiology/American Heart Association/Heart Rhythm Society AF guidelines (26), this cohort could be grouped as NVAF, which is in the absence of moderate-to-severe mitral stenosis or a mechanical heart valve. However, NVAF does not imply the absence of valvular heart disease. The evaluated heartvalves, rheumatic or artificial (EHRA) valve classification of patients with AF with VHD appears useful in categorizing these patients in terms of thromboembolism and bleeding risks (27). Meta-analysis-derived data from the original clinical trials suggest that, among patients with AF and various valvular defects and operations, DOACs reduce stroke and systemic embolism compared with warfarin, but with differences in bleeding risk (28), indicating that other valvular defects may break the balance of embolism and bleeding in a DOAC-dominant AF population. In our study, all the eight ischemic stroke (IS)/TE events occurred in patients without MR, which was consistent with the previous finding that aortic regurgitation and MR do not independently increase the thromboembolic risk beyond the AF alone and do not act as additional risk. In contrast, the bleeding risk seems more insidious in MR. Melggard et al. observed a 2.4–4.0% risk of major bleeding in this subgroup at 1 year after AF diagnosis (29). In the *post-hoc* subanalysis of the rivaroxaban once-daily, oral, direct factor Xa inhibition compared with vitamin K antagonism for prevention of stroke and embolism trial in atrial fibrillation (ROCKET-AF) AF trial, the rate of major bleeding was higher among patients with anticoagulated AF with aortic or MR compared to those without VHD (30). In our cohort, perioperative bleeding risk in all MR and mild/moderate MR was 14.8% and 19.0%, and even a trace MR would indicate an adjusted increasing risk (OR = 2.067, 95% CI 1.095–3.902, *p* = 0.025), with most of the events being minor bleeding. In comparison with Bisson's study in which patients with AF having MR had a higher CHA2DS2-VASc score but a similar risk of IS/thromboembolic than other patients with AF (31), our study found that patients with AF having MR had a similar HAS-BLED score but a higher risk of bleeding. It was indicated in the 1990's that MR might be protective against stroke, especially in those patients with left atrium (LA) enlargement (32). A general concept is that MR may play the role of a washing machine effect in the left atrium and might be associated with a lower risk of IS/thromboembolic events (31). Our study further indicated that MR, even at a trace level, served as a sensitive predictor in a more hypocoagulability state and was independently associated with perioperative bleeding. Additionally, the effect of MR appeared more significant in patients with higher HAS-BLED and CHA2DS2-VASc scores. For these patients who possibly need to be prescribed to OACs in a long term after CA, MR could serve as an additional risk marker for potential bleeding events.

The other factor independently associated with an increased risk of bleeding was a lower BMI. Obesity is a risk factor for all-cause and cardiovascular death, and despite this, an inverse relationship between overweight or obesity and a better cardiovascular prognosis in long-term follow-up studies has been observed (33). This phenomenon, named “obesity paradox,” has been reported in many AF studies consistent with ours (34, 35). The benefit of DOAC compared to warfarin across BMI categories is still debated. It seems like, compared to warfarin, DOACs were associated with better safety and effectiveness across all BMI categories, but preserved in patients who are underweight and patients with morbid obesity (36). In another meta-regression analysis comparing the effect of DOAC vs. warfarin across different BMI groups, the effect size advantage of DOACs compared with warfarin in terms of safety and efficacy gradually attenuated with increasing weight (37). In our cohort, the effect of BMI on perioperative bleeding was not different in both the warfarin and DOAC groups (p for interaction = 0.651), so in this DOAC-dominated anticoagulated cohort, the “protection effect” may largely be explained by a fixed dose of DOACs, leading to unintentional underdosing in patients who are obese or unintentional overdosing in patients who are underweight. Therefore, a weight-based dosage adjustment should be taken into consideration to achieve optimal benefits of DOACs for thromboembolic prevention in these patients with NVAF.

The latest meta-analysis evaluated different bleeding risk assessment tools to predict major bleeding events in patients with AF and concluded that HAS-BLED is a balanced bleeding risk assessment tool in terms of sensitivity and specificity, whereas the European score, ABC, and mOBRI are high-sensitivity tools and ORBIT, ATRIA, Shireman, and GARFIELD-AF are high-specificity tools (12), consistent with other systematic reviews and meta-analyses favoring the HAS-BLED score over others (11, 38). Another study showed that there was no long-term advantage of ABC-bleeding over the HAS-BLED score, whereas HAS-BLED was better in identifying patients with major bleeding events, mostly intracranial hemorrhage and gastrointestinal bleeding (16). Our cohort, with 96% having a HAS-BLED ≤ 2, compared different bleeding scores and found potential confounders in the anticoagulated Asian population of AF undergoing CA. Although the performance of the two bleeding scores was not different, the ABC-bleeding score had a moderate predictive value, of which the power did not reach statistical significance in the HAS-BLED score. Adding the most important factor, MR could increase the predictability of ABC score but not HAS-BLED score regarding C-indices. This “additional” sensitivity was also observed in patients with DOAC and a higher thromboembolic risk score, who were more likely to continue OAC therapy in the long run (not shown in tables). We also observed that the fully adjusted ABC model overestimated bleeding risk when the risk is higher, aligning with its innate sensitivity described before (12), and MR only predicted bleeding events in the ABC low-/moderate-risk group (Table 5). Therefore, a lower ABC score without MR could be intended for excluding bleeding risk in a postoperative population of AF who need long-term anticoagulation, while HAS-BLED combined with mild/moderate MR is more inclined to identify bleeding risk and indicate a more cautious use of anticoagulants during the postoperative period.

### Strengths and Limitations

To the best of our knowledge, we demonstrated the performance of additional clinical factors based on the most commonly used bleeding risk scores in an anticoagulated Asian population of AF undergoing CA for the first time. For patients with CHA2DS2-VASc score of 0 or 1 who initiate DOACs during the perioperative period and do not need long-term anticoagulation 2–3 months after CA, it is advised to adjust the duration or dosage of OACs based on MR status and BMI to avoid unnecessary risk of bleeding. Otherwise, for those who initiate and would possibly continue anticoagulation therapy in a long run based on their stroke risk profile, we found that MR was a stable predictor for bleeding, thus indicating those patients need closer follow-up for bleeding.

However, several limitations of this single-center, retrospective, nonrandomized study are noteworthy. First, the fact that patients who were selected to receive CA shared a less severe clinical condition and the comprehensive use of OACs led to a low rate of thromboembolic events limited the exploration of cofounders of thromboembolism. Second, due to the retrospective design, the exact degree of MR could not be quantitatively described. Due to the limitations of our study, the results should be interpreted with caution and need to be confirmed by findings from randomized controlled trials controlled by MR and BMI status.

## Conclusion

More severe MR and lower BMI are associated with a higher incidence of perioperative bleeding, which helps improve the predictability of increased individual bleeding risk of a patient with NVAF who has received CA therapy and OACs.

## Data Availability Statement

The original contributions presented in the study are included in the article/Supplementary Material, further inquiries can be directed to the corresponding author.

## Ethics Statement

The studies involving human participants were reviewed and approved by the Ethics Committee of Fuwai hospital. The patients/participants provided their written informed consent to participate in this study. Written informed consent was obtained from the individual(s) for the publication of any potentially identifiable images or data included in this article.

## Author Contributions

WH, MT, SZ, YJ, JC, and JW contributed to conception and design of the study. HH, HN, and XC organized the database. HH and CC performed the statistical analysis. HH and NZ wrote the first draft of the manuscript. CC and NZ wrote sections of the manuscript. All authors contributed to manuscript revision, read, and approved the submitted version.

## Conflict of Interest

The authors declare that the research was conducted in the absence of any commercial or financial relationships that could be construed as a potential conflict of interest.

## Publisher's Note

All claims expressed in this article are solely those of the authors and do not necessarily represent those of their affiliated organizations, or those of the publisher, the editors and the reviewers. Any product that may be evaluated in this article, or claim that may be made by its manufacturer, is not guaranteed or endorsed by the publisher.
