# Supplementary material for: Mitral Regurgitation and Body Mass Index Increase the Predictability of Perioperative Bleeding in Anticoagulated Patients With Nonvalvular Atrial Fibrillation
Source: Front Cardiovasc Med. 2022 Mar 28;9:846590. doi: 10.3389/fcvm.2022.846590 (PMC8996069; doi:10.3389/fcvm.2022.846590)
Supplement: Supplementary file 1 [file Data_Sheet_1.docx]

**Supplementary Table 1. Summary of bleeding events in the full cohort.**

| Bleeding events | No. of patients |
| --- | --- |
| Procedure-related bleeding |  |
| Pericardial effusion | 2 |
| Bleeding around puncture point | 12 |
| Procedure-unrelated bleeding |  |
| Intracranial hemorrhage | 0 |
| Dermorrhagia or mucosal hemorrhage | 49 |
| Gastrointestinal hemorrhage | 13 |
| Urogenital hemorrhage | 6 |
| Hemoptysis | 2 |
| Multi-site hemorrhage | 7 |

**Supplementary Table 2. Univariate and multivariate logistic regression model of possible indicators for procedure-unrelated or related bleeding events.**

| Characteristics | Procedure-unrelated bleeding | | Procedure-related bleeding | |
| --- | --- | --- | --- | --- |
|  | Unadjusted OR  (95% CI), P value | Adjusted OR  (95% CI), P value | Unadjusted OR  (95% CI), P value | Adjusted OR  (95% CI), P value |
| Age | 1.022 (0.995-1.049), 0.114 |  | 1.016 (0.961-1.075), 0.559 |  |
| BMI | **0.902 (0.832-0.978), 0.012** | **0.911 (0.840-0.989), 0.026** | 0.911 (0.769-1.080), 0.283 |  |
| MR |  |  |  |  |
| None | Reference | Reference | Reference | Reference |
| Trace | 1.910 (0.974-3.746), 0.060 | 1.783 (0.891-3.569), 0.102 | 3.237 (0.798-13.124), 0.100 | 2.188 (0.446-10.749), 0.335 |
| Mild-Moderate | **3.292 (1.164-5.040), 0.001** | **3.166 (1.612-6.217), 0.001** | **8.311 (2.477-27.888), 0.001** | **7.272 (2.137-24.739), 0.001** |
| P for trend | **0.001** | **0.002** | **0.003** | **0.006** |
| TR |  |  |  |  |
| None | Reference | Reference | Reference |  |
| Trace | 1.809 (0.963, 3.399), 0.065 |  | 1.391 (0.292-6.625), 0.678 |  |
| Mild-Moderate | 1.984 (0.895, 4.397), 0.091 |  | **5.523 (1.620-18.826), 0.006** |  |
| P for trend | 0.071 |  | **<0.001** |  |
| CrCl (ml/min) | 0.992 (0.982-1.002), 0.117 |  | 0.976 (0.952-1.000), 0.053 | 0.977 (0.952-1.003), 0.083 |
| NT-proBNP (pg/ml) | 1.000 (1.000-1.001) , 0.065 |  | 1.000 (0.999-1.001), 0.558 |  |
| hs-TnI (ng/l) | 1.001 (1.000-1.003), 0.063 | 1.001 (1.000-1.003), 0.146 | **1.002 (1.000-1.004), 0.022** | 1.002 (1.000-1.005), 0.060 |

Abbreviation as above mentioned

**Supplementary Table 3. C-indices, ROC curves comparison, IDI and NRI of HAS-BLED score and ABC-bleeding score with or without mitral regurgitation or BMI to predict procedure-unrelated bleeding in the full cohort.**

| Full cohort | C-index (95% CI) | P | NRI (%, 95%CI) | P | IDI (%, 95%CI) | P |
| --- | --- | --- | --- | --- | --- | --- |
| HAS-BLED | 0.56 (0.49-0.63) |  | Ref. |  | Ref. |  |
| HAS-BLED + MR**^†^** | 0.60 (0.53-0.68) | 0.143 | 35.45 (11.70-59.20) | **0.003** | 1.63 (0.55-2.70) | **0.003** |
| HAS-BLED + BMI**^†^** | 0.60 (0.53-0.67) | 0.245 | 21.79 (-2.84-46.43) | 0.083 | 0.65 (0.11-1.20) | **0.019** |
| HAS-BLED + MR + BMI**^†^** | 0.65 (0.59-0.72) | **0.009** | 49.06 (24.53-73.58) | **<0.001** | 2.04 (0.85-3.24) | **<0.001** |
|  |  |  |  |  |  |  |
| ABC**^†^** | 0.58 (0.51-0.65) | 0.541 | 8.71 (-16.13-33.55) | 0.492 | 1.25 (-0.90-3.39) | 0.255 |
| ABC + MR**^※^** | 0.64 (0.57-0.71) | 0.063 | 36.14 (12.39-59.88) | **0.003** | 1.73 (0.59-2.88) | **0.008** |
| ABC + BMI**^※^** | 0.62 (0.56-0.69) | 0.142 | 24.09 (-0.44-48.62) | 0.054 | 0.43 (-0.05-0.91) | 0.080 |
| ABC + MR + BMI**^※^** | 0.67 (0.61-0.73) | **0.011** | 44.01 (19.39-68.63) | **<0.001** | 1.62 (0.52-2.73) | **0.004** |

Data are c-indices, integrated discriminatory improvement (IDI) and net reclassification improvement (NRI) for each score and P value for their comparison with HAS-BLED score and ABC-bleeding score alone. † p for comparing with HAS-BLED score. ※p for comparing with ABC score.

Supplementary Table 4. Net benefit of using the HAS-BLED and ABC scores combined with MR and BMI or not for identifying perioperative bleedings conditional on different decision thresholds.

| Decision threshold (%) | Net benefit vs all negative | | | | |
| --- | --- | --- | --- | --- | --- |
|  | All positive | HAS-BLED | ABC | HASBLED+MR+BMI | ABC+MR+BMI |
| 4 | 0.521 | 0.521 | 0.526 | 0.541 | 0.508 |
| 6 | 0.266 | 0.266 | 0.253 | 0.346 | 0.415 |
| 8 | 0 | 0.076 | 0.15 | 0.178 | 0.203 |
| 10 | -0.278 | 0.057 | 0.054 | 0.177 | 0.076 |
| 12 | -0.568 | -0.008 | -0.006 | 0.077 | 0.073 |
| 14 | -0.872 | 0.007 | 0.007 | 0.104 | 0.04 |
| 16 | -1.19 | 0.006 | 0.014 | 0.042 | 0.029 |

Supplementary Table 5. Bleeding predictors between MR status and possible interaction.

|  | No/trace MR  (n=861) | Mild/moderate MR  (n=79) | P for  comparison | P for  interaction |
| --- | --- | --- | --- | --- |
| Age (years) | 57.7±9.8 | 61.2±9.1 | **0.003** | 0.499 |
| BMI (kg/m^2^) | 26.0±3.3 | 25.4±3.0 | 0.094 | 0.500 |
| CrCl (ml/min) | 88.2 (72.2, 105.1) | 77.1 (66.4, 92.9) | **0.003** | 0.748 |
| NT-proBNP (pg/ml) | 172.0 (61.5, 519.0) | 329.4 (150.8, 840.4) | **<0.001** | 0.227 |
| hs-TnI (ng/l) | 2.0 (0.0, 4.0) | 3.0 (2.0, 6.0) | **0.022** | 0.874 |
| Warfarin (%) | 124 (14.4) | 11 (13.9) | 0.534 | 0.334 |
| Echocardiography |  |  |  |  |
| LAD (mm) | 39.7±5.4 | 42.1±5.2 | **<0.001** | 0.410 |
| LVEDD (mm) | 47.9±4.5 | 49.2±6.0 | 0.066 | 0.267 |
| LVEF (%) | 62.3±5.6 | 61.3±6.6 | 0.133 | 0.963 |
| TR (mild/moderate, %) | 22 (2.6) | 48 (60.8) | **<0.001** | 0.181 |

Abbreviation as above mentioned.
